# Clinical and electrophysiological features of adult patients with combined central and peripheral demyelination- a systematic review

**DOI:** 10.3389/fimmu.2025.1572507

**Published:** 2025-06-04

**Authors:** Szymon Andrusiów, Edyta Dziadkowiak, Magdalena Koszewicz

**Affiliations:** ^1^ Clinical Department of Neurology, University Centre of Neurology and Neurosurgery, Wroclaw Medical University, Wroclaw, Poland; ^2^ Clinical Neurophysiology Laboratory, University Centre of Neurology and Neurosurgery, Faculty of Medicine, Wroclaw Medical University, Wroclaw, Poland

**Keywords:** combined central and peripheral demyelination (CCPD), myeloradiculoneuritis, encephalomyeloradiculoneuritis, neurofascin antibodies, lactosylceramide antibodies

## Abstract

**Background:**

The classification of combined central and peripheral demyelination (CCPD) is challenging due to unclear pathomechanisms and a lack of diagnostic and therapeutic criteria. Existing clinical data are limited to case reports or small series, with few attempts to define CCPD using radiological or molecular markers. Differential diagnosis depends on excluding well-characterized demyelinating diseases of the central and peripheral nervous systems. No systematic review has yet summarized the clinical, radiological, electrophysiological, molecular, and therapeutic evidence for CCPD.

**Methods:**

This review follows PRISMA (Preferred Reporting Items for Systematic Reviews and Meta-Analyses) guidelines, uses the JBI critical appraisal tool for case series and is registered at PROSPERO (CRD42025640575). A systematic search of Embase, MEDLINE, Web of Science, and Google Scholar was conducted for studies available up to December 2024. Inclusion criteria focused on adult patients with electrophysiological and imaging findings. Exclusion criteria included CCPD associated with infections, rheumatological conditions, or anti-MOG/anti-AQP4 antibodies.

**Results:**

Most patients exhibited hemiparesis assessed by MMT and MRC scales, with tetraparesis often asymmetrical. Imaging revealed either diffuse CNS involvement (cerebral hemispheres, brainstem, spinal cord) or lesions limited to one or two sites. Nerve conduction studies showed primarily demyelinating features. Treatment frequently involved combination therapies.

**Conclusions:**

This review underscores the dearth of high-quality data on CCPD, with extant studies frequently exhibiting a paucity of methodology for definitive analysis. The presence of elevated protein concentrations in CSF and the presence of antibodies, specifically anti-LacCer and anti-NF, has been identified as potential biomarkers of the disease. Furthermore, GCS in high doses might be one of the most effective treatment options.

**Systematic review registration:**

https://www.crd.york.ac.uk/PROSPERO/view/CRD42025640575, identifier CRD42025640575.

## 1 Introduction

In 1986, Amit et al. presented a case of a 10-year-old girl with simultaneous central and peripheral nervous system demyelination of acute onset and severe course, corresponding to the diagnosis of disseminated encephalomyelopathy and Guillain-Barré syndrome (1). In 1992 Amit et al. first used the term combined central and peripheral demyelination (CCPD) (2). The use of other terms for the co-occurrence of central and peripheral demyelination, such as relapsing-remitting disease of the central nervous system(CNS) and peripheral nervous system(PNS), peripheral neuropathy in the course of multiple sclerosis, and chronic inflammatory demyelinating polyneuropathy (CIDP) with central nervous system (CNS) involvement, points to the difficulty of the nosology. The occurrence of CCPD is very limited. Single cases or a small number of cases have been reported in the literature. To date, no guidelines have been developed to define diagnostic criteria and methods (1–3). However, Ogata et al. proposed to define CCPD by the following markers: on magnetic resonance imaging (MRI), visualization of high-intensity T2 signal changes in the brain, optic nerves, or spinal cord, or abnormalities on visual evoked potentials; and on nerve conduction studies, changes strongly supportive of the diagnosis of demyelination, such as reduction of conduction velocity, prolongation of F-wave latency, motor nerve conduction blocks, abnormal temporal dispersion. The exclusion of other demyelinating diseases was an additional criterion (4). In CCPD patients, Hou et al. suggested the determination of the following antibodies: MOG, AQP4, NF155, NF186, contactin 1 (CNTN1), CNTN2, contactin-associated protein-like 1 (CASPR1), CASPR2, MAG and neuronal cell adhesion molecule (NrCAM) (5).

The pathomechanism of CCPD is unclear. There is ambiguity as to whether the overlap between central and peripheral demyelination is coincidental or due to a common epitope in the central and peripheral nervous system (6). The other authors suggest a higher susceptibility to autoimmune disease (3). In the literature, CCPD patients have antibodies to neurofascin-155 (NF155), a protein expressed in both central and peripheral myelin, or to aquaporin-4 (AQP4) and, less frequently, to MOG (myelin-associated glycoprotein). Also, antibodies to MAG, a transmembrane glycoprotein located at the node of Ranvier in Schwann cells and oligodendrocytes, have been reported in patients with CCPD (5).

CCPD is a rare condition characterized by a varied clinical picture, often preceded by infection or vaccination (7). Most cases showed a progressive course rather than a relapsing-remitting or monophasic course (3). Clinical picture often suggests multiple sclerosis (MS) and CIDP in the same patient (8). In addition to optic neuritis, the most common symptoms are motor weakness, hyporeflexia, and sphincter dysfunction (3, 5).

In the case reports, corticosteroids (GCS) or intravenous immunoglobulin (IVIg) pulses were used as first-line therapy with good therapeutic efficacy. In cases with partial resolution of symptoms, other treatment options were tried. These included rituximab, plasmapheresis, and IFN-beta (3, 9, 10).

## 2 Materials and methods

This review was based on the PRISMA (Preferred Reporting Items for Systematic Reviews and Meta-Analyses) guidelines for systematic reviews (11), the JBI critical appraisal tool for case series studies (12) and was registered with PROSPERO (The International Prospective Register of Systematic Reviews) at number CRD42025640575.

### 2.1 Search strategy

The selection of databases searched was based on this study to optimize search strategies for publications for systematic reviews (13). After an initial review of the databases, it was decided to use keywords covering the widest possible range of publications and not to exclude publications due to the language used, due to the small amount of data available. Publications available as of December 2024 were searched in the following databases: Embase, MEDLINE, Web of Science and Google Scholar.

The following keywords were used: (‘combined central and peripheral demyelination’) NOT (‘multiple sclerosis’ OR ‘MS’ OR ‘neuromyelitis optica’ OR ‘anti-MOG’ OR ‘anti-AQP4’ OR ‘infection’ OR ‘Guillain-Barré syndrome’ OR ‘anti-ganglioside’). 125 search results were obtained, and after the rejection of 56 duplicates, 69 publications were included for further analysis. The publications that corresponded to the subject of the review were selected for further analysis, following an examination of their titles, abstracts and keywords. This analysis was conducted by two researchers (S.A. and E.D.). In the event of conflicting opinions, a third researcher (M.K.) was consulted for a third opinion. At this stage, 21 publications that were not related to the subject, four review papers, one animal experimental paper and two pediatric studies were rejected. Furthermore, 12 records were rejected as they were Conference Abstracts. Subsequently, six publications were rejected due to unavailability of the full text. In the subsequent stage, two investigators assessed the full texts of the articles for meeting the inclusion and exclusion criteria, and for quality, according to the JBI critical appraisal tool for case series studies (12). Publications written in a language other than Polish or English were analyzed using the tool DeepL (14). Three studies were excluded due to the inclusion of a pediatric population in the study group and the inability to extract adult population data. In addition, 12 studies were rejected due to poor quality, primarily gaps in clinical description and lack of included electrophysiological results. In addition, one further publication that was not found in the databases was included in the review during the citation search, after the publication had been checked for quality. Finally, nine case-studies were included in the review.

**Figure 1:**
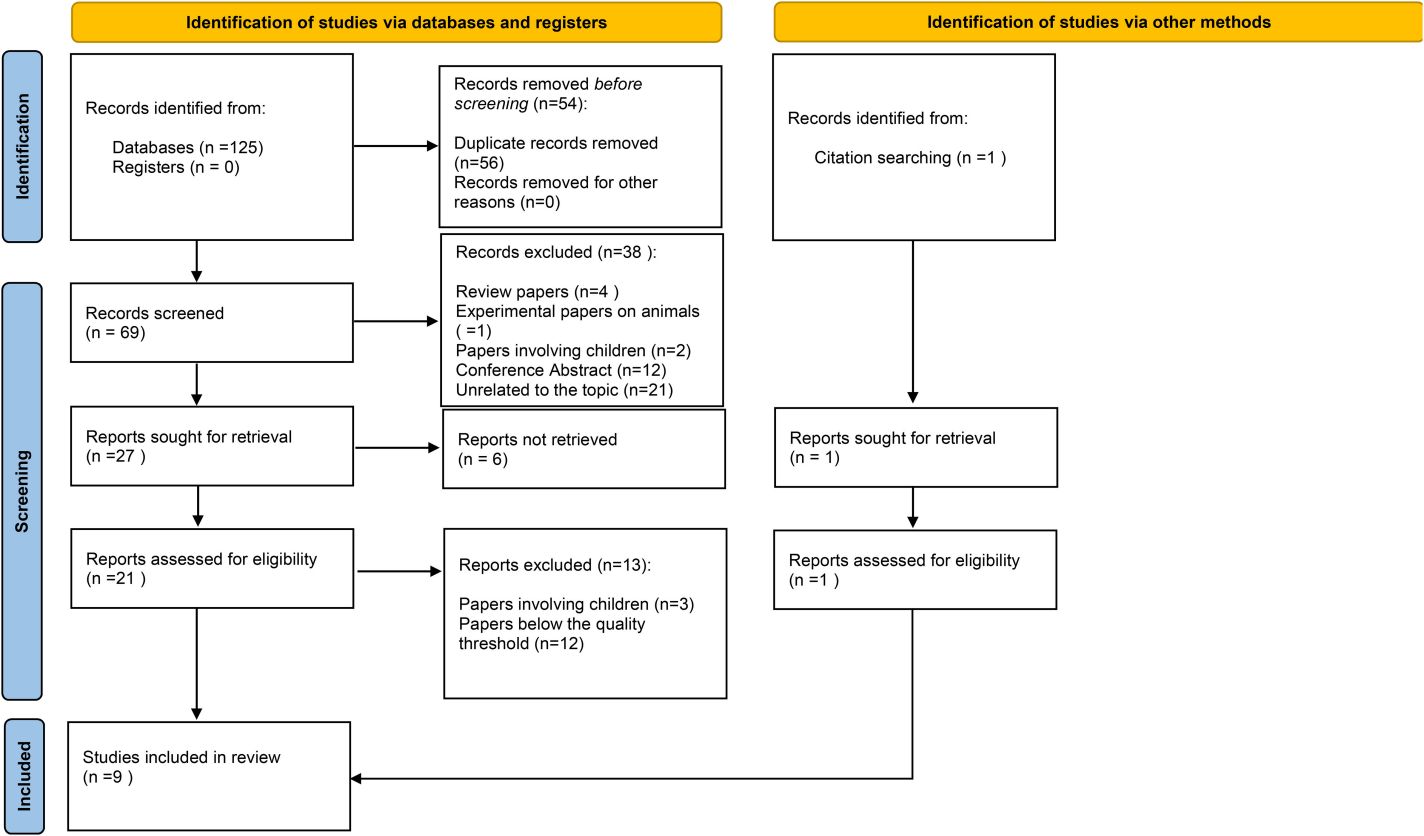
The publication search strategy (Prisma 2020 Flow Diagram).

The publication search strategy and following steps are shown on the Prisma 2020 Flow Diagram (**Figure 1**).

### 2.2 Inclusion and exclusion criteria

Inclusion criteria were: (1) the study group evaluates the adult population; (2) the clinical description includes electrophysiological and imaging findings; (3) the publications meet the quality assessment criteria according to the JBI critical appraisal tool for case series studies (12).

Exclusion criteria included: (1) studies evaluating pediatric populations; (2) studies describing the coexistence of CCPD with infections or rheumatological diseases; (3) publications describing the coexistence of central and peripheral lesions in the course of disease with anti-MOG and anti-AQP4 antibodies, due to the known clinical and pathogenesis of these disease entities; (4) review publications that provide only summary data without the possibility to access the electrophysiological findings; (5) guideline publications; (6) experimental animal or human studies that do not adequately describe the clinical data of the study group.

### 2.3 Data extraction and quality assessment procedure

Data extraction (**Table 1**) by the researchers consisted of collecting information on the cases described, such as nationality, age of onset, sex, co-morbidities and clinical course, including symptoms of PNS (motor and sensory) and CNS involvement, time since onset of the above, MRI and NCS findings and laboratory tests, including antibody determinations and CSF tests, therapies used, their efficacy and recurrence.

**Table 1:** Characteristics of the studies analyzed.

Study	Country	Gender	Age at onset	CNS/PNS sign interval	Motor symptoms	Sensory symptoms	CNS symptoms	Magnetic resonance imaging (Brain/Spinal Cord)	NCS/EMG results	Conduction velocity (m/s)	CSF analysis	Antibodies	Treatment	Relapses	Comorbidities	Other information
Saito et al., 2018 (16)	Japan	Male	45	2 months (first PNS)	Asymmetric paresis in upper limbs (3/5 in MRC)	Paresthesia in lower limb	Abducens nerve palsy Dysphagia	Bilateral medulla oblongata, right side of pons, white matter of the right temporal lobe (T2-hyperintense) Right temporal lobe (T1 Gd+)	1. Dispersed compound muscle action potentials 2. Absence of F waves	MCV ulnar 25/19 MCV median 9/15 MCV tibial posterior NR SCV median 29/23 SCV ulnar 42/NR SCV sural NR	Protein 46 mg/dL Pleocytosis 64/mm 3(mono 100%)	anti-lactosylceramide (anti-LacCer) (serum, CSF)	1. IVIg 0,4g/kg/day for 5 days- not effective. 2. Methylprednisolone i.v 3g- effective. 3. Methylprednisolone i.v 3g followed by the oral prednisolone (1mg/kg/day)- effective	2	None	
Menon et al., 2014 (17)	India	Male	52	0	Tetraparesis more proximal (2/5 in MRC) Neck flexor paresis	None	Dysarthria Ptosis Disorders of consciousness (drowsy)	Central pontine myelinolysis (T2-hyperintense)	1. Prolonged of F waves 2. SCV correct	MCV median 25 MCV tibial posterior 30 MCV peroneal 41	Not performed (contraindications to lumbar puncture)	None	Methylprednisolone (high dose)- effective	None	Chronic autoimmune hepatitis (ANA and ASMA positive)	
Moodley et al., 2024 (18)	South Africa	Female	Late 20s 1 month post partum	0	Asymmetric tetraparesis (4/5 in MRC) Diaphragmatic paresis	Glove and stocking superficial and deep sensory impairment Lower limbs paresthesia	Headaches Visual acuity impairment with swollen optic discs (bilateral) Prolonged P100 latencies bilateral in VES	White matter of bilateral hemispheres, splenium, corpus callosum genu (T2-hyperintense)	1. Reduced SNAPs 2. Conduction Blocks 3. Temporal dispersion 4. Prolonged of F waves 5. No active denervation in EMG	MCV ulnar 29/31 MCV median 26/28 SCV sural NR	Protein 97 mg/dL Oligoclonal bands t. 2	None	1. IVIg for 5 days + azathioprine 150mg/d + oral GCS-partial effective (in vision, not in motor symptoms) 2. Mycophenolate mofetil 3g/d + oral GCS- effective 3. IVIg + Methylprednisolone- not effective	1 (dead)	None	Sisters In whole-exome sequencing: missense variant in PPFIA4 and a nonsense mutation in CHCHD10
		Female	Early 20s 2 weeks post partum	0	Asymmetric tetraparesis (4/5 in MRC) Bilateral facial weakness	Glove and stocking superficial and deep sensory impairment	Disorientation to time and place Prolonged P100 latencies bilateral in VES	White matter of bilateral hemispheres, splenium, corpus callosum genu (T2- hyperintense)	1. Absent SNAPs in 4 limbs 2. Conduction Blocks 3. Temporal dispersion 4. Prolonged of F waves 5. Active denervation in EMG	MCV ulnar 21/26 MCV median 28/31 SCV sural NR	Protein 128 mg/dL Pleocytosis 8/mm 3(mono 100%) Oligoclonal bands t. 2	None	1. IVIg + oral GCS 60mg/d + azathioprine 100 mg/d- effective	None	HIV infected 2 years after CCPD	Sisters In whole-exome sequencing: missense variant in PPFIA4 and a nonsense mutation in CHCHD10
Hoshino et al., 2017 (19)	Japan	Male	59	3 months (first CNS)	Asymmetric tetraparesis (lower limbs 1/5 in MMT, upper limbs 4/5)	Superficial and deep sensor impairment (all limbs) Upper limbs paresthesia	Diplopia Dysarthria Dysphagia	Medulla oblongata (T2-hyperintense) Cervical(C 4-5) and thoracic(Th 6/Th10) central grey matter and nerve roots (T1 Gd+ and Th2-hyperintense)	1. Prolonged of F waves	MCV ulnar 48/46 MCV median 47/45 MCV tibial posterior 37/37 MCV peroneal 32/30 SCV median 48/43 SCV ulnar 46/47 SCV sural 48/47	Protein 137 mg/dL Pleocytosis 36/mm3(mono 100%) Oligoclonal bands MBP 1 pg/mL CXCL 486 pg/mL	anti-galactocerebroside (anti-Gal-C)(serum) anti-LacCer (serum)	Methylprednisolone i.v 3g (5 times)- effective.	None	Hypertension Dyslipidaemia Diabetes	Dysuria
Nomura et al., 2021 (20)	Japan	Male	62	9 years (first CNS)	Left limbs hemiparesis Gait disturbance	Asymmetric paresthesia (lower limbs and right hand)	Recurrent optic neuritis	Cervical (C 3-6) and thoracic (Th 4-5) spinal cord (T2- hyperintense)	1. Reduced CMAPs in tibial nerves bilateral	MCV upper limbs 33 lower limbs 33 SCV upper limbs 32 lower limbs 44	Protein 206 mg/dL MBP 136 pg/mL	None	GCS pulse 1g/d (2 times)- effective	None	None	Recurrent optic neuritis, 4 times from 62 years old
Harada et al., 2019 (21)	Japan	Female	60	2 months (first PNS)	Tetraparesis (lower limbs 2/5 in MMT, upper limbs 3/5 in MMT)	None	Babinski sign bilateral Sensory level with paresthesia at Th6	Medulla oblongata and cervical/thoracic spinal cord (T1 Gd+) Medulla oblongata and cervical/thoracic spinal cord, medullary cone and cauda equine (T2-hyperintense)	1. Reduced CMAPs in peroneal nerves bilateral 2. Conduction block in the tibial nerve 3. Absence of F waves in peroneal nerves	MCV ulnar 68 MCV median 60 MCV tibial posterior 40/40 MCV peroneal 41/47 SCV median 49 SCV ulnar 53 SCV sural 46/NR	Protein 83 mg/dL Pleocytosis 35/mm3(mono 100%) Oligoclonal bands MBP 818 pg/mL	anti-LacCer (serum)	1. Methylprednisolone 3g, (2 times)- not effective 2. Selective plasma exchange + Methylprednisolone g- low effective 3. Total plasma exchange+ oral prednisolone 20mg/d- effective 4. Methylprednisolone 3g (2 times) + 40mg oral prednisolone – effective (at relapse)	1	None	Constipation
Nonaka et al., 2015 (22)	Japan	Male	18	5 years (first CNS)	Tetraparesis (4/5 in MMT) Gait disurbance Pes cavus	Superficial and deep sensor impairment (all limbs) Paresthesia (all limbs)	Speech impairment Spastic paraparesis Feet clonus Babinski sign bilateral Vision loss in right eye Prolonged P100 latencies bilateral in VES	Periventricular white matter, pons, medulla oblongata, cerebellum, thoracic spinal cord (T2- hyperintense)	1. Reduced CMAPs	MCV ulnar 30 MCV median 31 MCV tibial posterior 23 MCV peroneal 24 SCV median NR SCV ulnar NR SCV sural NR	Protein 96 mg/dL Olgigoclonal band (in relapse)	anti-NF	1. Methylprednisolone 3g (2 times) + prednisolone 20mg/d-effective 2. IFN-β1b- not effective	1	None	
Shimizu et al., 2016 (23)	Japan	Male	21	Unclear	Gait disturbance	Superficial and deep sensor impairment (distal lower limbs) Paresthesia (all limbs)	None	Right frontal lobe white matter (T2-hyperintense) Nerve roots hypertrophy in lumbar level (T1 Gd+)	1. Distal motor latency prolonged in the tibial nerves 2. Prolonged of F waves in ulnar and median nerves bilateral 3. Time dispertion in motor nerves 4. Reduced SNAP amplitudes	MCV 30	Protein 542 mg/dL Pleocytosis 11/mm3 (mono 55%)	anti-NF155	1. IVIg 0,4mg/kg/d+ 2 times 3x1g GCS i.v. + 20mg/d prednisolone- not effective 2. Plasma exchange-effective 3. Cyclosporin 100 mg/d (at relapse)- unclear	1	None	Positional hand tremor
Thomas et al., 1987 (24)	Great Britain	Male	28	6 years (first CNS)	Tetraparesis (distal) Gait disturbance	Superficial and deep sensory impairment (distal) Paresthesia (all limbs)	Recurrent optic neuritis Sensory level at Th10 Prolonged P100 latencies in VES	Periventricular (Spin Echo)		MCV ulnar 30 MCV median 16 MCV peroneal 24 SCV median NR SCV ulnar NR SCV sural NR	Protein 280 mg/dL	None	1. ACTH 2. Prednisolone 3. Azathiopryne 100 mg/d All partial effective	12	None	Multiple autonomic symptoms Back and abdomen pain
		Female	21	Unclear (first PNS)	Tetraparesis Gait disturbance	Superficial and deep sensory impairment (distal) Paresthesia (all limbs)	Vision impairment	Periventricular (Spin Echo)	1. Reduced SNAP amplitudes	MCV ulnar 15 MCV median 35 MCV peroneal 18	Protein 44 mg/dL	None	1. ACTH-partial effective 2. ACTH- partial effective 3. Prednisolone + Azathioprine 100mg/d-effective	7	None	Multiple autonomic symptoms
		Male	33	3 years (first CNS)	Tetraparesis (distal) Gait disturbance	Superficial and deep sensory impairment (distal)	Headache Vision impairment Diplopia Internuclear ophthalmoplegia Spastic paraparesis Dysarthria	Brainstem, Cerebellar, Periventricular (Spin Echo)		MCV ulnar 27 MCV median 28 MCV peroneal NR SCV median NR SCV ulnar NR SCV sural NR	Protein 428 mg/dL	None	1. ACTH- not effective 2. Prednisolone 60mg/d + Azathioprine 3mg/kg/d- effective	6	None	
		Male	38	1 year (first PNS)	Tetraparesis Feet drop Gait disturbance Fasciculations	Superficial and deep sensory impairment (in feet) Paresthesia (upper limbs) Pain, allodynia (mainly feet)	Vision impairment Dysarthria Pseudobulbar affect Internuclear ophthalmoplegia	Brainstem, Cerebellar, Periventricular (Spin Echo)		MCV ulnar 15 MCV median 11 SCV median NR SCV ulnar NRF SCV sural NR	Protein 250 mg/dL	None	1. Azathioprine 100mg/d- partial effective	3	None	Action tremor Ataxia
		Male	41	Unclear	Tetraparesis	Superficial and deep sensory impairment (asymmetric)	Diplopia Vision impairment Internuclear ophthalmoplegia Facial weakness Mild cognitive impairment Spastic asymmetric paresis	Not performed	1. Reduced SNAP amplitudes	MCV ulnar 25 MCV median 31 MCV peroneal 23	Protein 225 mg/dL Oligoclonal bands	None	Unclear	3	None	Ataxia
		Female	31	Unclear	Tetraparesis	Superficial and deep sensory impairment (distal)	Optic neuritis Dizziness Diplopia Dysarthria Spasti paraparesis (mild) Prolonged P100 latencies in VES	Brainstem, Cerebellar, Periventricular (Spin Echo)		MCV ulnar 14 MCV median 17 MCV peroneal NR SCV median NR SCV ulnar NR SCV sural NR	Protein 173 mg/dL	None	1. ACTH- effective 2. Azathioprine 2,5mg/kg/d- not effective 3. Prednisolone 60mg/d- not effective	Unclear		Ataxia Epilepsy

Articles were selected by two researchers independently using the JBI critical appraisal tool for case series protocol (12). Each study was assessed for the following characteristics: clear inclusion criteria, clear method of measuring the condition, valid methods in the diagnostic process, consecutive and complete inclusion of participants in the series, clear reporting of demographic and clinical data, detailed reporting of treatment methods and outcomes. It was decided not to consider the assessment of the demographic structure of the study center setting and methods of statistical analysis. Each characteristic could be rated as Yes/No/Unclear/Not applicable. If any attribute was assigned ‘No’, the study was excluded from the review.

### 2.4 Data coding

Data coding was performed by two investigators. Age at onset was defined as the time of onset of first symptoms as determined by history or, in the case of acute onset, age at first hospitalization. As the onset of symptoms (acute/chronic) was unclear in many of the cases analyzed, this parameter was not analyzed. If the nationality of the patient was not clearly defined, it was assumed to be the same as the nationality of the main author of the study. The main motor symptoms analyzed were the presence and distribution of paresis and its severity on clinical scales (MRC or MMT). Due to the compatibility of these scales, they were analyzed together in further analyses. Sensory symptoms were coded in terms of negative and positive symptoms and their distribution, excluding sensory disturbances with a sensory level likely to be related to spinal injury. Coding for symptoms of CNS involvement was based on the diagnostic criteria described by Wang et al. (15) and included changes in MRI and visual evoked potentials (VEPs). Because of the possibility of similar symptoms with involvement of the cranial nerves (PNS) or their nuclei in the brainstem (CNS), the assignment of symptoms to a particular column in the table depended on the results of additional tests (MRI or VEP). Symptoms for which the origin (CNS or PNS) could not be clearly explained have been placed in the “Other information” column. The presence of MRI lesions was coded on the basis of the study description in the publications and the accompanying images. The location of imaging lesions on T2-dependent sequences and the presence of contrast enhancement after gadolinium (Gd) administration on T1-dependent sequences (T1 Gd+) were recorded. Abnormal nerve conduction study (NCS) or electromyography (EMG) results were coded in the NCS/EMG results column. If the authors provided motor nerve conduction velocity (MCV) and/or sensory nerve conduction velocity (SCV) values, these were recorded separately. Non-response was coded as ‘NR’. Several studies provided extensive information with specific conduction velocity values of multiple nerves; in such cases, if a nerve was assessed bilaterally, both values were given after ‘/’. NCS results and CSF laboratory values were rounded to whole numbers. Only CSF parameters with abnormal values were coded. If the authors reported the location of the antibodies (CSF or serum), this was recorded. If different therapies were used consecutively, this was indicated by sequential numbers, and specific drug doses were coded if the authors made this clear. Effective therapies were defined as those that resulted in resolution or stabilization of symptoms leading to cessation of hospitalization. Relapse was defined as recurrence of symptoms or new MR/NCS lesions after an asymptomatic period or after a period of symptom stabilization requiring a change in treatment. In addition, any other relevant information that did not fit into the other columns was recorded in the remaining columns.

## 3 Results

The majority of studies were from Japan (67%) (16, 19–23), with one each from India (11%) (17) South Africa (11%) (18) and Great Britain (11%) (24). Two studies were a case series of two patients (18, 24) the others were case reports. Of the 15 patients included in the review, the majority were male (67%) (16, 17, 19, 20, 22–24). Onset of symptoms ranged from 18 to 62 years of age. Six patients developed symptoms before the age of 30 years (18, 22–24), and the remaining six patients developed symptoms after the age of 40 years (16, 17, 19–21, 24). In three patients, symptoms of peripheral and central nervous system (PNS and CNS) involvement occurred simultaneously (17, 18). In four patients, the disease started with PNS involvement (16, 21, 24) and the interval between symptoms was two months to one year. In contrast, in five patients, the disease began with symptoms of CNS involvement, with a longer duration of interval between symptoms which was three months to nine years (19, 20, 22, 24). Two patients had comorbidities: one suffered from autoimmune hepatitis (17) and the other had multiple cardiovascular risk factors (hypertension, dyslipidemia, diabetes) (19).

Symptoms of PNS involvement ranged from mild in six cases (17, 18, 21, 22, 24) to severe (preventing independent walking) in the remaining cases. Most patients were assessed for paresis using the MMT and MRC scales, which were considered equivalent. Paresis was present in 14 patients (93%), most commonly as tetraparesis in 12 patients (17–19, 21, 22, 24) which was asymmetric in five cases (17–19, 21). Several patients differed from the general clinical picture: one presented proximal paresis and neck muscle involvement (17), one bilateral facial weakness (18), and one presented only gait disturbances without paresis (23). Thirteen patients clinically presented sensory disturbances, mainly in the form of paresthesia of varying severity in nine cases (16, 18, 19, 22–24), in two patients, these were the only sensory symptoms (16, 20). In the remaining 11 patients, sensory impairment of varying severity was predominantly distributed distally. Clinical signs of PNS correlated with changes in NCS in 10 cases. In the remaining five patients, NCS changes did not correlate with the severity of clinical symptoms, where in four patients the conduction was significantly worse than the clinical signs (18, 22, 23) while in one patient the clinical signs were more severe than the conduction changes. NCS findings were predominantly demyelinating in nature. Reduced conduction velocities were found in 14 patients (16–20, 22–24) and conduction blocks in three cases (18, 21). Decreased SNAP/CMAP or no response, most likely axonal lesions secondary to demyelination, was seen in 12 cases (18, 20–24). In summary, severe/moderate nerve conduction changes were described in 13 patients (16–18, 20, 22–24).

CNS symptoms included brainstem damage in eight cases (16, 17, 19, 22–24) encephalopathy in two (18), optic nerve damage in another seven (18, 20, 22, 24) and myelopathy in four (20–22, 24). One patient, without CNS symptoms, had asymptomatic frontal lobe lesions on MRI (23). All patients showed demyelinating lesions on MRI. On imaging, lesions in one case showed diffuse CNS involvement (22), involving the cerebral hemispheres, brainstem and spinal cord. In a further six patients, lesions were confined to two sites (brainstem and spinal cord or cerebral hemispheres and brainstem) (16, 19, 21, 24). In the remaining seven patients, lesions were found in a single location: in the brainstem with a type of central myelinolysis of the bridge (17), in the cerebral hemispheres (18, 23, 24) and in the spinal cord (20). Spinal cord lesions were seen at the cervical and/or thoracic levels in four cases (19–22), one of which also had lesions in the medullary cone and cauda equina (21).

The clinical course in most cases was relapsing-remitting, with at least one relapse observed in 10 patients (16, 18, 21–24). In two patients the relapse occurred during the reduction of oral prednisolone (21, 22).

CSF biochemistry showed changes in 13 patients. Twelve patients had elevated protein levels ranging from a minimum of 83 to a maximum of 542 mg/dl (18–24). In five cases pleocytosis was observed, of which four had pleocytosis exclusively of mononuclears (16, 18, 19, 21) and one (10%) consisted of mononuclears in 55% (23). Six patients had oligoclonal bands (18, 19, 21, 22, 24) including three with moderate/severe NCS changes (18, 22), and another three with myelopathy (19, 21, 22). In addition, three publications assessed other parameters in the CSF and found elevated levels of MBP protein, with concentrations ranging from 1–818 pg/mL (19–21), and one publication described elevated CXCL levels of 486 pg/mL (19). Anti-LacCer (16, 19, 21) and anti-NF (22, 23), antibodies were detected in the serum of five patients, and two types of antibodies, anti-LacCer and anti-Gal-C, were detected in one patient. Patients with anti-LacCer were older (group >40 years), had pleocytosis on PMR, two of them had oligoclonal bands (19, 21) and all had post-contrast enhancing MRI lesions and brainstem involvement.

Several treatment methods were used: IVIg, plasmapheresis, GCS (ACTH, oral prednisolone, intravenous methylprednisolone), other oral immunosuppressive drugs (azathioprine, cyclosporin, mycophenolate mofetil, IFN-β1b) and combinations of the above methods. In nine patients, several lines of treatment were necessary due to low efficacy and relapses (16, 18, 21–24). GCS in various forms were used in 14 patients as first-line or subsequent treatments. The use of GCS was associated with a significant improvement in the clinical condition of nine patients (16–22, 24). The therapy of high-dose pulses (3 days of 1g each) of methylprednisolone, repeated up to five times, were effective in six patients (16, 17, 19–22). In three patients the above-mentioned therapy was ineffective (18, 21, 23). One patient, resistant to methylprednisolone during the first hospitalization (when remission was achieved after plasmapheresis), responded to this treatment during a relapse of symptoms (21). Four patients were treated with IVIg (alone or in combination therapy) (16, 18, 23) in one patient the therapy was effective (18), in the other one patient the therapy was partially effective (18), in the remaining two patients the therapy was ineffective (16, 23). Total plasma exchange was used in two patients (21, 23) and in both cases the therapy was effective. Other therapies were effective in treating four patients, but always in combination with GCS (18, 24).

Two publications describe cases that differ from the others (18, 22). In the case of the two sisters described in the case series (18), the acute onset of symptoms was related to pregnancy, occurring in one, two weeks and in the other one, a month after delivery. The clinical course was similar in both patients: moderate-severe peripheral sensorimotor syndrome with moderate/severe changes on neuroconduction studies (NCS), mainly of the sensory nerves, conduction blocks and signs of encephalopathy (headache, disorientation), optic nerve involvement and diffuse changes in the cerebral hemispheres and corpus callosum (visible on MRI). One patient died during a relapse (probably from respiratory failure due to involvement of the diaphragm muscle), which was the only death in the group (7% mortality rate). In the second patient, the disease was monophasic, which may have been related to the acquisition of HIV infection and the introduction of antiretroviral therapy. In both cases, WES detected mutations: a missense variant in the PPFIA4 gene and a nonsense mutation in the CHCHD10 gene. In the case of one patient (22), the authors of this review suggested a chronic, possibly congenital nature of the disorder, based on clinical symptoms (pes cavus) and disproportionately large NCS lesions, which did not correlate with the severity of clinical symptoms.

## 4 Discussion

This systematic review of case reports highlights the paucity of good quality data on CCPD. During the course of the review, the authors of this publication came across more extensive studies, but their methodology did not allow an unambiguous analysis of CCPD. Large observational studies conducted to date have included the pediatric population (4, 15, 25) or have evaluated patients with anti-MOG or anti-AQP4 antibodies together (5, 10, 15) who have a well-defined clinical picture. In our opinion, this may have distorted the clinical data of adult patients with CCPD as a separate nosological entity with a pathophysiology distinct from NMOSD or MOGAD. However, as demonstrated by the authors of the above observational studies, CCPD is likely to be characterized by a wide range of clinical manifestations and a diverse course and prognosis, but is arguably an entity with a worse prognosis than CIDP or MS, as we have also demonstrated in this review. Differential diagnosis is even more challenging when considering the similarity of lesion characteristics on imaging studies in patients with different demyelinating diseases. Kasaab (26) showed that there were no significant differences in nerve ultrasonography between patients with CIDP or CCPD; he also found abnormalities in 30% of patients with MS, which may further complicate the differential diagnosis. Cortese et al. (27) divided patients with CCPD into smaller subgroups with different clinical pictures: myeloradiculoneuritis, encephalomyeloradiculoneuritis, and a further subgroup whose clinical picture corresponded to MS with CIDP. These observations are consistent with the results of this review and may indicate a different pathophysiology (perhaps different pathological antibodies) in these subgroups of patients with CCPD. As we have shown in this review and in light of the above, the diagnosis of CCPD requires a thorough analysis of symptoms and a long follow-up of patients. The review uses the diagnostic criteria proposed by Wang et al. (15), which are a refinement of those previously developed by Ogata et al. (4). The authors of the review evaluated the included articles in terms of their compliance with the above criteria. In some cases, changes suggesting CNS or PNS demyelination were minor, but sufficient in the authors’ opinion. For example, in the article by Shimizu et al. (23) minor changes in the CNS were observed, but due to their characteristics – asymmetry of location, patient age, clinical picture – they were considered highly likely to be demyelinating in nature. Similarly, the article by Hoshino et al. (19) treats minor changes, this time in the PNS, which the authors classified as likely demyelinating due to meeting the EFNS/PNS criteria and the overall clinical picture. The authors are aware of the limitations of the currently available diagnostic criteria, which allow for considerable freedom in interpreting the results of additional tests, but require extensive differential diagnosis. Ogata et al. (4) show a high prevalence of anti-NF155 antibodies (45.5%), which is not confirmed by a study of a European population (Italy, France) by Cortese et al. (27). As suggested by Kira (28), the prevalence of anti-NF155 antibodies may be related to the specific haplotype HLA-DRB1*15:01-DQB1*06:02 that she showed in the Japanese population. This may be a reason for the lower prevalence of anti-NF155 in the European population, but further research is needed. The pathological picture of CCPD has been reported by many authors in the presence of anti-LacCer, anti-GalCer and anti-glucosylceramide (GlcCer) antibodies (16, 21, 29–32). MBP protein, mentioned in the review as a potential biomarker, is unfortunately a non-specific marker of CNS damage and as such probably has little relevance in the differential diagnosis of CCPD (33). In contrast, markedly elevated protein levels in the CSF may be an indication to expand the diagnosis towards CCPD, as we have shown in this review.

Molecular mimicry mechanisms may be involved in the initiation of the autoimmune response in CCPD. There are reports of disease following vaccination or infection, including COVID-19 (7, 34–38) and rheumatological co-morbidities (39). This review clearly shows the greatest efficacy of high doses of intravenous GCS as the best therapeutic option, which is also confirmed by other authors (4, 5, 25, 15). Plasmapheresis is also mentioned as a possible therapeutic option (4). In all the above-mentioned studies, IVIg showed the least efficacy, which is consistent with our results. In addition, some authors propose rituximab in refractory cases and show its high efficacy (5, 40). However, due to the heterogeneous study groups and the lack of large studies outside the Japanese and Chinese populations, the above conclusions should be verified by multicenter studies in a larger population.

## 5 Conclusions

The development of clear guidelines for the diagnosis and treatment of CCPD will require multicenter studies evaluating large patient populations. In designing such guidelines, it is suggested that patients with positive anti-AQP4 or anti-MOG be excluded, and that adult and pediatric populations be assessed separately.

Clinical manifestations of CCPD can include the entire spectrum of CNS and PNS symptoms, ranging from asymptomatic findings in supportive investigations to severe, life-threatening symptoms. Therefore, the authors suggest considering the diagnosis of CCPD in a wide range of patients, especially those with demyelinating neuropathies and central lesions of unclear etiology. In cases of suspected CCPD, MRI of the brain and cervical spine appears to be the minimum investigation. In addition, the testing for anti-LacCer and anti-NF antibodies, and perhaps anti-Gal-C, which may be potential markers of the disease, seems reasonable. Extended diagnostics seems most justified in patients with very high levels of protein in CSF.

It is conceivable that genetic testing for a missense variant in the PPFIA4 gene and a nonsense mutation in the CHCHD10 gene may emerge as a future marker for the onset of CCPD symptoms in young adults; however, given the dearth of literature on the subject, further studies are required to substantiate this claim.

Based on all available data, the authors of this review suppose that high-dose GCS treatment and possibly plasmapheresis may have certain advantages over other therapeutic options. In addition, we recommend considering prolonged maintenance treatment and monitoring (NCS/MRI) due to frequent relapses.

## Data Availability

The original contributions presented in the study are included in the article/supplementary material. Further inquiries can be directed to the corresponding author.
